# A new approach to an old problem: Overview of the East African Community’s Medicines Regulatory Harmonization initiative

**DOI:** 10.1371/journal.pmed.1003099

**Published:** 2020-08-12

**Authors:** Margareth Ndomondo-Sigonda, Gugu Mahlangu, Margaret Agama-Anyetei, Emer Cooke

**Affiliations:** 1 African Union Development Agency–New Partnership for Africa’s Development, Midrand, South Africa; 2 Medicines Control Authority, Harare, Zimbabwe; 3 African Union Commission, Addis Ababa, Ethiopia; 4 World Health Organization, Geneva, Switzerland

**Summary points**

- Substandard and/or falsified medical products may result in treatment failure and/or death.
- The capacity to regulate medical products is key for ensuring the quality, safety, and efficacy of medical products circulating in a market.
- The experience of the East African Community, during its piloting of the Medicines Regulatory Harmonization initiative, serves as a lesson for scaling up the African Medicines Regulatory Harmonization Program across the continent.
- Currently, the East African Community’s Medicines Regulatory Harmonization initiative is poised to transition from a donor-funded pilot project into a self-sustaining, permanent feature of the African regulatory landscape.
- Government, partner, and public support is needed for strong systems to regulate medicines in the East African Community and elsewhere in the world.

This paper is an overview of the Special Collection in *PLOS Medicine* on the East African Community (EAC)’s Medicines Regulatory Harmonization (MRH) initiative, which seeks to improve the region’s capacity to regulate medical products and increase patient access to quality, safe, and efficacious medical products. This paper also reflects on the future of this initiative within the context of the African MRH Program and as a foundation for the African Medicines Agency (AMA), which was adopted by the Heads of State and Governments of the African Union (AU) and is expected to be established in the next few years.

To illustrate the problems caused by weak regulation of medical products in Africa, we draw readers’ attention to the case of a 13-year old boy from Kampala, Uganda, treated for bacterial meningitis at Mulago National Referral Hospital in 2013 [1]. Doctors promptly began treating him with ceftriaxone, but on the patient’s tenth day in the hospital, he died. Afterwards, the vial of ceftriaxone used was found to contain less than half the amount of the medicine stated on the label. This patient’s death, which was likely caused by substandard medicine, is a tragedy; unfortunately, it is not a rare one.

Similar stories unfold every day in sub-Saharan Africa, where roughly 10% of medicine samples—including of lifesaving medicines such as antimalarials, antibiotics, and antiretrovirals—are either substandard or falsified [2]. Even higher percentages of poor-quality medicines are found in some African countries with poorly functioning regulatory authorities [3–5]. In 2013 alone, an estimated 122,350 children under 5 years of age living in sub-Saharan Africa died as a result of poor-quality antimalarials [6]. Weak regulatory systems and infrastructure allow medicines that do not meet internationally acceptable standards of safety, quality, and efficacy to circulate in a market and subsequently be used. Fixing this problem requires National Medicines Regulatory Authorities (NMRAs) to have the necessary political, public, and government support; legal mandate to undertake regulatory functions; access to adequate human and financial resources; and proper management and enforcement systems.

## Creation of the EAC MRH initiative

**Fig 1:**
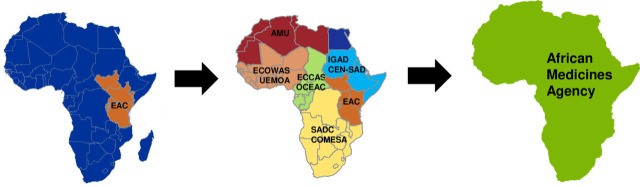
The EAC MRH initiative now exists alongside similar initiatives started by other African Union–recognized regional economic communities as part of the AMRH Program. Ultimately, the goal of the AMRH is to support the establishment of an African Medicines Agency that will improve access to quality medicines across the continent [10]. AMRH, African Medicines Regulatory Harmonization; AMU, Arab Maghreb Union; CEN-SAD, Community of Sahel-Saharan States; COMESA, Common Market for Eastern and Southern Africa; EAC, East African Community; ECCAS, Economic Community of Central African States; ECOWAS, Economic Community of West African States; IGAD, Intergovernmental Authority on Development; MRH, Medicines Regulatory Harmonization; OCEAC, Organization of Coordination for the Fight Against Endemic Diseases in Central Africa; SADC, South African Development Community; UEMOA, Union Economique et Monétaire Ouest Africaine. *Credit for map modified by authors*: *Boston Consulting Group*.

The EAC, a regional economic community composed of Burundi, Kenya, Rwanda, South Sudan, Tanzania, and Uganda (Fig 1), is no stranger to the problem of poor-quality medicines. In a 2011 survey, 25% of respondents from Kenya reported that either they or members of their households had been victims of fake medicines; the percentages were 24% in Tanzania and 18% in Uganda [7]. With the goal of improving its citizens’ access to safe and effective medicines, and ending stories like the aforementioned one, the EAC launched its MRH initiative in 2012. The initiative’s purpose was to increase access to quality medicines throughout the EAC by harmonizing technical standards and optimizing regulatory requirements and oversight processes for medicines [8,9]. With support from partners including the AU Development Agency–New Partnership for Africa’s Development, World Bank, World Health Organization, Swiss Agency for Therapeutic Products (Swissmedic), United Kingdom Department for International Development, and Bill & Melinda Gates Foundation, the initiative provided the needed technical, financial, and political advocacy support.

As a result, countries in the region have developed shared regulatory requirements and standards for medical products regulation, dramatically simplifying multistate applications for users and allowing Partner States to begin conducting joint regulatory activities. The initiative has also helped create new NMRAs in Rwanda, South Sudan, and Zanzibar. Furthermore, the initiative piloted a process that has reduced the average time it takes for NMRAs to register new medicines by roughly half. In the coming years, the initiative will build on these achievements while continuing to expand into other regulatory functions as well.

We believe that sharing information about the EAC MRH initiative with the greater stakeholder community is important because the program’s overarching goal—to improve people’s access to quality medicines—is shared by many other regions of the world. Indeed, the right to a standard of living adequate for health is enshrined in the Universal Declaration of Human Rights [11], and given the importance of medicines to health, United Nations Sustainable Development Goal 3.8 calls for access to “safe, effective, quality and affordable essential medicines and vaccines for all” [12]. The importance of regulatory harmonization and cooperation in ensuring access to quality versions of essential medicines is well recognized [13]. Therefore, in this Special Collection of 5 articles, we describe the EAC MRH initiative from its beginning through its plans for the future.

Sharing information about the EAC MRH initiative is also important because it represents the first step on a path leading toward continent-wide, coordinated regulation of medicines. Since the EAC MRH initiative started, a number of other regional economic communities have launched their own regional harmonization initiatives. In total, these initiatives now cover more than 85% of the continent (Fig 1) [14]. Each of these initiatives has unique elements, reflecting the views of the regional economic community that created it; however, at the heart of each regional approach are the fundamentals of relying on the work products of others to help inform one’s own regulatory decisions and on work sharing among NMRAs to best utilize available human and financial resources. The story of the EAC MRH initiative is the first (and the most advanced) of many stories about regulatory standards harmonization and process optimization in Africa, each of which will have great value as the AMA continues to take shape.

The AMA is expected to become the continental focus of regulatory standards harmonization, process optimization, and resource coordination across Africa. In February 2019, the treaty establishing the AMA was endorsed by the heads of government of the AU. In order for the AMA to become a reality, the parliaments of 15 AU member states must ratify the treaty, which is expected to take another 2 to 3 years. Although the final form that this new regulatory body will take is unclear at present, it is expected to draw on the best aspects of Africa’s existing regional initiatives, as well as other initiatives that have established regional and continental regulatory processes [10].

The story of the EAC MRH initiative may have value outside of Africa as well. In establishing the program, the EAC has been forced to contend with challenges that will be familiar to many regions in other parts of the world. For example, its NMRAs have very different levels of regulatory maturity. Many are grappling with underfunding, understaffing, and markets that do not command the same attention as larger markets elsewhere in the world. When crafting the initiative, the EAC was aware that it could not merely copy the structure of existing regional regulatory bodies, which had developed over the course of decades; instead, it needed to develop a structure that could immediately confer benefits while acknowledging the political and economic realities of the region. Thus, rather than relying on a single regional regulatory body, the EAC MRH initiative relies on joint assessment decisions made by Partner States’ NMRAs, informed by data gathered from joint activities. The willingness of Partner States to rely on joint assessments when making national registration decisions is driven by trust, goodwill, and respect for expertise, rather than legal requirements. The initiative also emphasizes work sharing, with each Partner State providing expertise and leadership in a different regulatory domain; this is one way of keeping every NMRA involved while leveraging the limited expertise across the region. Although this voluntary and decentralized arrangement is well suited to the local context, it also creates organizational challenges, which will be discussed in the other articles in the Collection. Some of the innovative ideas generated by the initiative, described in greater detail in the articles that follow, may be useful to other regional regulatory bodies seeking to pool resources and collaborate in order to improve health [8].

The present article serves as an introduction to the Collection. It is our hope that each paper in this Special Collection will provide information about the EAC MRH initiative that the larger regulatory community can use to continue moving toward greater harmonization of regulatory standards and optimization of regulatory processes.

- In the first paper of the Collection [15], individuals who have been involved with the program since the beginning explain how the initiative started.
- The second paper [16] provides the perspective of the people who helped turn a vision of greater optimization of medicines regulation and improved harmonization of technical standards across the EAC into a reality. It discusses the program’s accomplishments thus far, as well as the challenges the initiative has encountered.
- The individuals tasked with shaping the program’s next phase describe their plans for the coming years in the third paper [17].
- Finally, in the fourth paper [18], regulatory experts from around the world help place this program’s work in a broader context, with the hope that this will help other regulatory harmonization and optimization programs learn from one another’s experiences.

## Future perspectives

Today, the EAC MRH initiative is poised to transition from a donor-funded pilot project into a self-sustaining, permanent feature of the African regulatory landscape, as discussed in the third paper in the Collection [17], helping to provide the engine for developing the new AMA. We anticipate that the AMA will build on the initiative’s accomplishments to advance regulatory harmonization across the African continent. In turn, the AMA may shape future regional initiatives. As the EAC MRH initiative continues to improve its ability to conduct core regulatory activities, it will also begin to pursue some ambitious new goals, such as establishing a semiautonomous regional regulatory body—the EAC Medicines Agency—by 2022. We look forward to seeing how the EAC MRH initiative grows and changes over time and observing the shape that other regional initiatives around the world take as they too identify paths toward improving access to quality versions of essential medicines.

## Abbreviations

AMA: African Medicines
AgencyAU: African Union
EAC: East African Community
MRH: Medicines Regulatory Harmonization
NMRA: National Medicines Regulatory Authority
Swissmedic: Swiss Agency for Therapeutic Products
